# Mendelian randomization study of telomere length and lung cancer risk in East Asian population

**DOI:** 10.1002/cam4.2590

**Published:** 2019-10-11

**Authors:** Xuguang Cao, Mingtao Huang, Meng Zhu, Rui Fang, Zijian Ma, Tao Jiang, Juncheng Dai, Hongxia Ma, Guangfu Jin, Hongbing Shen, Jiangbo Du, Lin Xu, Zhibin Hu

**Affiliations:** ^1^ Department of Epidemiology School of Public Health Nanjing Medical University Nanjing China; ^2^ Department of Thoracic Surgery Nanjing Medical University Affiliated Cancer Hospital Nanjing China; ^3^ Department of Thoracic and Cardiovascular Surgery First People's Hospital of Yancheng Yancheng China; ^4^ Center for Global Health School of Public Health Nanjing Medical University Nanjing China; ^5^ Jiangsu Key Lab of Cancer Biomarkers, Prevention and Treatment Collaborative Innovation Center for Cancer Medicine Nanjing Medical University Nanjing China

**Keywords:** Asian population, lung cancer, mendelian randomization, telomere length

## Abstract

Associations between telomere length and cancer risk have been investigated in many epidemiological studies, but the results are controversial. These associations may be biased by reverse causation or confounded by environmental exposures. To avoid potential biases, we used Mendelian randomization method to evaluate whether TL is the causal risk factor for lung cancer. We conducted Mendelian randomization analysis in two published East Asian GWAS studies (7127 cases and 6818 controls). We used both weighted genetic risk score and inverse‐variance weighting method to estimate the relationship between TL and lung cancer risk. Nonlinear test also used to detect potential association trends. We observed that increased weight GRS was associated with increased risk of lung cancer (OR = 2.25, 95%CI: 1.81‐2.78, *P* = 1.18 × 10^−13^). In different subtypes, weight GRS was significantly associated with lung adenocarcinoma risk (OR = 2.69, 95% CI: 2.11‐3.42, *P* = 7.20 × 10^−16^); while lung squamous cell carcinoma showed a marginal association (OR = 1.45, 95% CI = 1.01‐2.10, *P* = .047). Nonlinear analysis suggested a log‐linear dose‐response relationship between increased weight GRS and lung cancer risk. Our results indicated that longer TL increases lung cancer risk. Those biological mechanisms changes caused by long TL may play an important role in lung carcinogenesis.

## Graphical abstract

Telomere length GRS indicated that longer TL increases LC risk. A log linear dose‐response relationship was observed between increased GRS and LC risk.

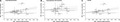

## 1 INTRODUCTION

Lung cancer is the most common malignant tumor worldwide, accounting for 11.6% of all diagnosed cancer and 18.4% of all cancer deaths in 2018.^1^ Consistent with world trends, lung cancer remains the most common cancer in Chinese population, as well as the leading cause of cancer‐related death.^2^ Tobacco smoking is the major risk factor of lung cancer, and approximately 90% cases can be attributed to smoking.^3^ Also, genetic factors play an important role in lung cancer carcinogenesis. In the past decade, genome‐wide association studies (GWAS) successfully identified lots of lung cancer susceptibility loci, such as *CHRNA5*‐*CHRNA3*‐*CHRNB4* region of chromosome 15q25 and human leukocyte antigen region. Even so, at least 25% of lung cancer cases were never smokers and the heritability of lung cancer was also relatively low, nearly 18%.^4^, ^5^, ^6^, ^7^ The risk factors of lung cancer still need more exploration.

Telomeres, located at the end of each chromosome, are specialized DNA‐protein structures and play essential roles for life functions. Human telomeres consist of long tracts of double‐stranded TTAGGG repeats. During DNA replication, telomeres would prevent the ends of chromosomes shortening and help keep genome stability and integrity.^8^, ^9^ Previous studies show that telomere length (TL) may be a double‐edged sword. Short TL may lead to genetic instability, as well as cellular senescence and apoptosis.^10^ Long TL or telomerase activity up regulation may promote cell growth and proliferation.^11^, ^12^ Thus, telomeres are crucial in human carcinogenesis. A number of studies suggest that TL was associated with multiple cancer types, but associations are not consistent because of the dual role of telomeres in carcinogenesis. For example, many prospective studies measuring TL in peripheral leukocytes showed that lung cancer risk increased with longer telomeres.^13^, ^14^, ^15^, ^16^ However, a large prospective study based on Danish population found there was no association between TL and lung cancer.^17^

Because of the limitation of observational study, the true relationship between TL and cancer risk may be obscured by confounding factors, such as age at TL measurement. Mendelian randomization (MR), based on the random assortment of genetic variants during meiosis, is an effective method to test the causal effect in observational studies. MR uses instrumental variables to evaluate the relationship between the exposure and an outcome.^18^, ^19^ In MR analysis, using genetic variants associated with certain exposure or phenotype as instrumental variables can avoid potential confounding bias. Previous lung cancer MR studies also suggested increased lung cancer risk was associated with long leukocyte TL.^20^, ^21^ However, those studies were either in Western population or in East Asian never‐smoking women.

To elucidate causal effects for lung cancer risk, we conducted an MR method and selected genetic variants significantly associated with TL as instrumental variables to estimate the causal relationship between TL and lung cancer risk in a pooled East Asian population.

## 2 MATERIALS AND METHODS

### 2.1 Study subjects

The pool samples included two previous published lung cancer GWAS studies from East Asia. The details of subjects were described in the original studies.^22^, ^23^, ^24^ A total of 13 945 samples (7127 cases and 6818 controls) were enrolled from our previous Chinese population lung cancer GWAS study (NJMU, 5408 samples from China) and published GWAS from the Female Lung Cancer Consortium in Asia (FLCCA, 8537 samples from East Asia).^22^, ^23^, ^24^ There are 4773 lung adenocarcinoma cases and 1482 lung squamous cell carcinoma cases in pooled samples. The rest 872 lung cancer cases were classified in other histology types. Each study obtained informed consent from the participants and was approved by the respective Institutional Review Boards. The detailed information for all samples is shown in Table S1.

### 2.2 Genetic instrumental variables selection

We used TL‐related SNPs as MR instrumental variables. SNPs were selected from previously published TL GWAS studies, following these criteria: (a) reported SNP signals showed genome‐wide association significance level with TL (*P* ≤ 5×10^−8^); (b) minor allele frequency (MAF) for TL‐related SNPs more than 0.05 in East Asian population; (c) variants having low linkage disequilibrium (LD) between each SNP (*r* ^2^ < .5). MAF and LD information was calculated from the 1000 Genomes Project (Phase 3) ASN subjects. Finally, we chose nine SNPs identified in leukocyte TL GWAS and met selection criteria for further analysis.^25^, ^26^, ^27^ Based on previous studies, we obtained long TL allele as effect allele, as well as association estimate for the long allele (in terms of kb increase in TL per allele). Details for nine SNPs used in our studies are list in Table S2.

### 2.3 Quality control and genotype imputation

Quality control and genotype imputation for two GWAS studies have been fully discussed in previous articles.^22^, ^23^, ^24^ In brief, genotyping in NJMU data used Affymetrix Genome‐Wide Human SNP Array 6.0 chips. The FLCCA data was obtained from public database (the database of Genotypes and Phenotypes, Study Accession: phs000716.v1.p1) and genotyping was conducted in Illumina 610Q SNP microarray and Illumina 660W SNP microarray. For standing quality control procedures, we first used PLINK software (v1.90) to exclude low quality individuals and low quality SNPs. Samples with low call rates, extreme heterozygosity rates and familial relationships, as well as SNPs with low call rates, low MAF, and violating the Hardy‐Weinberg equilibrium, were all removed. The overlapped samples in FLCCA and NJMU GWAS data were excluded from the FLCCA GWAS samples. GWAS data imputation was performed by IMPUTE2 software (v2.3.2) using 1000 Genomes Project Phase 3 data as an imputation reference.

### 2.4 Statistical analysis

We applied two MR methods based on individual level and summary statistic level, respectively. Firstly, we used the nine selected TL‐associated SNPs to build genetic predicted leukocyte telomere length. We calculated weighted genetic risk scores (GRS) using the following formula:

$${\text{GRS}}_{i}=\sum_{j=1}^{9}\beta_{j}x_{\mathrm{ij}}$$

Here, *x* represents the number of long alleles for the *j* ^th^ SNP in the *i* ^th^ subject (*x_ij_* = 0, 1 or 2) and *β_j_* is the weight for the *j* ^th^ SNP. All weights were obtained from published TL GWAS studies (Table S2) and scaled to kb of TL per long allele to uniform weight scale. Finally, we used weighted GRS to predict individual telomere length, like an instrumental variable. We performed logistic regression to estimate the association between weighted GRS and lung cancer risk, adjusting for age, sex, pack‐years, first principal component, and different study. In addition to weighted GRS approach, we also used another summary data based MR method called inverse‐variance weighting (IVW) method to evaluate the association for TL and risk of lung cancer. This method has been fully described by Burgess et al^28^ and has been successfully used in many studies. In this study, we used the same nine SNPs’ summary statistics to estimate potential causal effects of TL. IVW method was conducted by “gtx” package (v0.0.8) in R software (v3.3.1). We also used aggregate test, which used log likelihood ratio test to compare a null model only including covariates with a model having all TL‐associated SNPs and all covariates, to calculate the total effect of all TL‐associated SNPs.

To better investigate the effect of TL on lung cancer risk, we categorized weighted GRS into 10 groups based on its decile distribution in all participants and tested association in each group to observe trends. What's more, we further used a restricted cubic spline analysis to examine whether there were potential nonlinear trends between TL and lung cancer risk.

### 2.5 Sensitivity analyses

For a causal interpretation of MR, instrumental variables need to meet several important assumptions. First, instrumental variables are associated with the exposure; Second, instrumental variables can affect the outcome only via the exposure; Third, instrumental variables are not associated with any confounders of the exposure‐outcome association.^29^ Violations of MR assumptions may lead to unreliable results. Since all the nine SNPs included in this analysis were significantly associated with leukocytes TL, which meet the first MR assumption. We further test if there is any violation of the rest assumptions. Under the second and third assumptions, TL‐related SNPs’ effect on TL should be proportional to their effect on lung cancer risk. We used “gtx” package pleiotropy test function to assess the second and third assumptions.

All statistical analyses were performed using PLINK (v1.90) and R (v3.3.1). Two‐sided *P *< .05 was considered statistically significant.

## 3 RESULTS

### 3.1 Association estimates for individual SNPs with LC

The associations between nine TL‐related SNPs and lung cancer risk in all participants was described in Table 1, suggesting that most of the TL‐related SNPs were not in observed significant association with lung cancer, except rs2736100 and rs10936599. Meanwhile, except rs2736100 and rs11125529, the rest seven SNPs did not show significant heterogeneity between two datasets (Table S3). Associations with *P* < .05 were found for lung adenocarcinoma (rs10936599, rs2736100), lung squamous cell carcinoma (rs2736100, rs7675998, rs755017). Aggregate test showed one or more TL‐related variants were in relation to lung cancer risk in aggregate (pooled *P* < 1×10^−8^). The results of NJMU and FLCCA data were similar with the overall results (Table S3).

**Table 1:** Associations of telomere length‐associated variants and lung cancer risk

SNP	Lung adenocarcinoma			Lung squamous cell carcinoma			Overall		
	OR	95%CI	*P* e	OR	95%CI	*P*	OR	95%CI	*P*
rs10936599	1.12	(1.06,1.19)	2.42 × 10^−5^	1.05	(0.96,1.14)	.300	1.10	(1.04,1.15)	2.51 × 10^−4^
rs2736100	1.37	(1.30,1.45)	5.17 × 10^−30^	1.21	(1.11,1.32)	1.25 × 10^−5^	1.33	(1.26,1.39)	5.05 × 10^−29^
rs7675998	1.06	(0.98,1.14)	.126	1.13	(1.01,1.27)	.034	1.07	(1.00,1.14)	.052
rs4387287	0.99	(0.92,1.06)	.708	0.98	(0.87,1.10)	.694	0.99	(0.93,1.06)	.795
rs8105767	1.05	(0.99,1.11)	.131	0.97	(0.89,1.06)	.523	1.02	(0.97,1.08)	.385
rs755017	0.99	(0.94,1.05)	.809	0.89	(0.82,0.97)	8.44 × 10^−3^	0.97	(0.93,1.02)	.297
rs11125529	1.05	(0.98,1.13)	.157	1.06	(0.95,1.18)	.317	1.05	(0.99,1.12)	.120
rs3027234	1.07	(0.93,1.23)	.368	1.04	(0.83,1.30)	.729	1.05	(0.92,1.18)	.482
rs412658	1.03	(0.97,1.09)	.367	0.99	(0.91,1.08)	.809	1.03	(0.97,1.08)	.331
Aggregate test^a^			3.43 × 10^−29^			7.79 × 10^−5^			1.51 × 10^−27^
Genetic risk score^b^	2.69	(2.11,3.42)	7.20 × 10^−16^	1.45	(1.01,2.10)	.047	2.25	(1.81,2.78)	1.18 × 10^−13^
MR(IVW)^c^	2.82	(2.21,3.61)	1.32 × 10^−16^	1.51	(1.03,2.22)	.037	2.37	(1.90,2.96)	1.85 × 10^−14^
Heterogeneity^d^			1.57 × 10^−15^			3.23 × 10^−4^			6.55 × 10^−16^

a Aggregate test is a log likelihood ratio test comparing a model having all telomere length‐associated SNPs and covariates with a null model.

b Genetic risk score ORs refer to a 1‐kb increase in telomere length.

c Inverse‐variance weighted Mendelian randomization estimate for a 1‐kb increase in telomere length.

d Pleiotropy test for significant heterogeneity across the nine SNP instruments used in the Mendelian randomization analysis.

e *P* value was from logistic regression adjusting for age, sex, pack‐years, and first principal component.

### 3.2 MR estimates based on weight GRS

**Figure 1:**
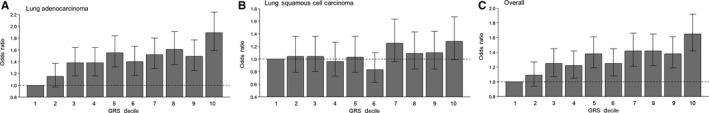
ORs for each telomere length‐associated GRS decile by lung adenocarcinoma (A), lung squamous cell carcinoma (B), and lung cancer overall (C). The lowest GRS decile is used as the reference of comparison

We then examined the relationship between the weighted GRS and lung cancer risk. Adjusting for potential confounders, we found that increased weighted GRS was associated with increased lung cancer risk (OR = 2.25, 95% CI: 1.81‐2.78, *P* = 1.18 × 10^−13^, Table 1). In subgroup analysis of lung cancer, weighted GRS was also significantly associated with lung adenocarcinoma (OR = 2.69, 95% CI: 2.11‐3.42, *P* = 7.20 × 10^−16^); while in lung squamous cell carcinoma, weighted GRS showed a marginal association (OR = 1.45, 95% CI = 1.01‐2.10, *P* = .047). Consistent results were also observed when analyzing two studies independently (Table S3). The decile of TL‐associated weight GRS result shows per decile increase in weighted GRS was significantly associated with increased risk of lung adenocarcinoma (OR = 1.05, 95% CI = 1.04‐1.07, *P* = 6.04 × 10^−14^), lung squamous cell carcinoma (OR = 1.02, 95% CI = 1.00‐1.04, *P* = .039), and combined lung cancer (OR = 1.04, 95% CI = 1.03‐1.06, *P* = 9.48 × 10^−13^). In lung adenocarcinoma type, compared with the first GRS decile, we found upper GRS deciles were in relation to increased lung cancer risk and we also observed a general rising trend. However, we did not observe any clear trend in lung squamous cell carcinoma (Figure 1).

### 3.3 MR estimates based on summary data

**Figure 2:**
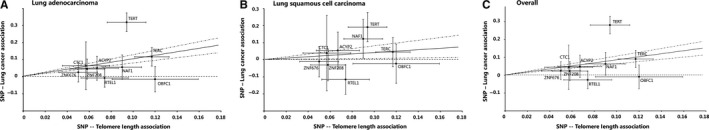
Scatter plot of the effect of each variant on telomere length and lung adenocarcinoma (A), lung squamous cell carcinoma (B), and lung cancer overall (C). Scatter plots show the per‐allele association with lung cancer risk plotted against the per‐allele association with kb of TL (with vertical and horizontal black lines showing 95% CI for each SNP). The scatter plot is overlaid with the Mendelian randomization estimate (slope of solid line with dashed lines showing 95% CI) of the effect of TL on lung cancer risk

The MR analysis based on summary data using the IVW method showed almost similar effect estimates with weighted GRS method (Table 1).The associations in NJMU GWAS and FLCCA GWAS were consistent with pooled participants (Table S3). Figure 2 showed all nine SNPs’ per long allele association with lung cancer risk, including two subtypes, plotted against the per long allele effect with kb of TL (vertical and horizontal black lines showing 95% CI for each SNP). The effects of TL on lung cancer were displayed as solid red lines with slopes meaning the MR estimates (dashed lines showing 95% CI). We found positive slopes in all lung cancers as well as in two histology subtypes, indicating that longer TL showed a significant positive association with lung cancer risk.

### 3.4 Nonlinear associations test between weight GRS and lung cancer

**Figure 3:**
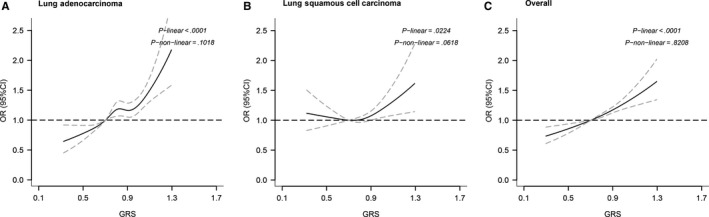
Nonlinear test between genetically increased TL and lung squamous cell carcinoma (A), lung adenocarcinoma (B), and lung cancer overall (C), based on restricted cubic spline function in the logistic regression model

In the analysis of the decile of TL‐associated GRS, we found an approximately log‐linear relationship between GRS and risk of lung cancer (Figure 1). Since “U shape” associations have been found in several studies, we used a restricted cubic spline analysis to fit the model to further investigate the liner trend between weight GRS and lung cancer risk. As shown in Figure 3, a significant log‐linear association was found in combined lung cancers subtypes (*P*‐linear < .001; *P*‐nonlinear = .821). In two subtypes of lung cancer, lung adenocarcinoma also demonstrated a significant log‐linear association (*P*‐linear < .001; *P*‐nonlinear = .102). However, lung squamous cell carcinoma showed a marginal nonlinear association (*P*‐linear = .022; *P*‐nonlinear = .062) between GRS and lung squamous cell carcinoma risk.

### 3.5 Sensitivity analysis

In pleiotropy test (Table 1), we found that lung adenocarcinoma, lung squamous cell carcinoma and combined lung cancer, all showed a significant deviation from MR assumptions two and three (*P* for pleiotropy = 1.57 × 10^−15^, 3.23 × 10^−4^ and 6.55 × 10^−16^, respectively). It indicated that one or more TL‐related SNPs’ effects on TL were not proportional to their effects on lung cancer risk. When testing of the pleiotropic effect for each SNP, rs2736100 in the TERT region was only one that failed in the test. SNP rs2736100 was a known lung cancer risk SNP reported in many studies across different population, and it may affect lung cancer risk through different mechanisms instead of TL. After excluding SNP rs2736100 (Table 2), we can still find significant associations between TL‐related weight GRS and increased risk of lung adenocarcinoma (OR = 1.66, 95% CI = 1.27‐2.16, *P* = 1.65 × 10^−4^) and combined lung cancer (OR = 1.45, 95% CI = 1.15‐1.84, *P* = 1.70 × 10^−3^). However, there was no significant association between lung squamous cell carcinoma and weight GRS (OR = 1.06, 95% CI = 0.71‐1.58, *P* = .784). The IVW method showed the same results. Besides, no significant evidence for pleiotropy was found in all lung cancer (*P* = .091) and lung adenocarcinoma (*P* = .116), and lung squamous cell carcinoma showed a marginal pleiotropy effect (*P* = .049) (Table 2). In separate analyses for each study, we found that without rs2736100, the association between TL‐related weight GRS and lung cancer risk was only significant in FLCCA data (Table S3). However, we also found there was no significant heterogeneity between two studies. The fewer samples of NJMU data may limit detection of the association. When excluding pleiotropic SNP rs2736100, the TL‐related variants met all MR assumptions and the association between GRS and lung cancer (including lung adenocarcinoma) remained statistically significant suggesting that TL may be a causal factor for lung cancer risk.

**Table 2:** Associations of telomere length‐associated variants and lung cancer risk after excluding rs2736100

SNP	Lung adenocarcinoma			Lung squamous cell carcinoma			Overall		
	OR	95%CI	*P*	OR	95%CI	*P*	OR	95%CI	*P*
Genetic risk score exclude rs2736100^a^	1.66	(1.27,2.16)	1.65 × 10^−4^	1.06	(0.71,1.58)	.784	1.45	(1.15,1.84)	1.70 × 10^−3^
MR(IVW)exclude rs2736100^b^	1.70	(1.30,2.23)	1.26 × 10^−4^	1.06	(0.69,1.62)	.795	1.50	(1.18,1.91)	1.12 × 10^−3^
Heterogeneity^c^			.116			.048			.091

a Genetic risk score ORs refer to a 1‐kb increase in telomere length after excluding rs2736100.

b Inverse variance weighted Mendelian randomization estimate for a 1‐kb increase in telomere length after excluding rs2736100.

c Test for significant heterogeneity across the nine SNP instruments used in the Mendelian randomization analysis after excluding rs2736100.

## 4 DISCUSSION

In this study, we conducted a telomere length MR studies in East Asian population. Considering advantage of MR method, we can avoid confounding bias and estimate the causal relationship between telomere length and lung cancer risk. We found that longer TL showed positive association with increased lung cancer risk. After sensitivity analyses, positive association in lung adenocarcinoma was still significant. Using restricted cubic spline analysis, we observed a linear relationship between genetic predicted TL and the risk of lung cancer. We also validated the results in two studies independently and did not find significant heterogeneity, suggesting a reliable association result in East Asian population.

Leukocyte TL and lung cancer risk relationship have been investigated in many previous studies. Several retrospective case‐control studies reported negative associations between TL and lung cancer risk. For example, Jang JS et al found that individuals with short telomeres were at a significant higher risk of lung cancer than those with long telomeres in 243 lung cancer cases and 243 healthy controls.^30^ With small sample size and TL measuring on diagnosed cancer participants, those studies may be misled by reverse causation bias. Prospective studies with large sample size observed longer TL increased the risk of lung cancer in multiple populations.^13^, ^14^, ^15^, ^16^ However, another large prospective study including 47 102 participants found no significant association between TL and lung cancer risk.^17^ The previous inconsistent finding may be attributed to small sample size, confounding factors, such as age at TL measurement and accuracy of TL assessment.^16^ Using MR method, potential confounding bias may be avoided by choosing genetic variants which are significantly associated with TL as instrumental variables. A MR analysis of TL using multi‐SNP score in European population observed a significant association between long telomeres and lung adenocarcinoma (but not squamous cell carcinoma), which is accordance with our results.

The main function of telomeres is to maintain chromosome integrity and stability during cell division. Because of the dual role of telomeres in tumor development, the relationship between TL and the risk of cancer is still unclear.^12^, ^31^ Short telomeres could result in replicative senescence and apoptosis and may act as tumor suppressors. Contrarily, long telomeres may allow for extra cell division, which let cells have more chances to accumulate carcinogenesis somatic mutations, and finally resulted in malignant transformation.^32^, ^33^ In previous melanoma and B‐cell lymphoma studies, researchers found that long TL was associated with increased cancer risk.^34^, ^35^ It is suggested that long telomeres may have a stronger effect than short telomeres in carcinogenesis, with a proposed mechanism that long telomeres may promote cell growth and proliferation, thus delaying senescence and allowing further oncogenic mutations to accumulate. In the nonlinear test, we did find a significant linear trend in lung cancer, which meant increased GRS, presenting longer telomeres, was associated with increased lung cancer risk and the risk rose linearly. Together with other studies, we support that long telomeres are a risk factor of lung cancer.

Given its advantages, MR approach becomes an effective and reliable method for investigated relationships between TL and lung cancer risk. Genetic instrument shows its own advantage, that is, genetic risk score is more stable than other risk factors, considering genetic sequence is constant during whole life time.^36^ MR approach would not be influenced by confounding bias or reverse causation, because TL estimation is based on germline level and individual's genetic predicted TL exists before lung cancer. Moreover, after sensitivity analysis, no pleiotropic effects for genetic variants remained, which met the second and third assumptions. However, there are still some limitations in our study. Just like other studies using SNPs as instrumental variables, SNPs only explained small phenotype variance. Considering variance in measured TL explained by SNPs is approximately 1%,^37^ we may lose some power to detect the causal effects. Nevertheless, previous studies also used a few SNPs as surrogate measures of peripheral leukocyte TL and found significant association results.^21^, ^38^ In addition, we used leukocyte TL instead of TL from lung tissues due to lack of lung tissue‐specific TL GWAS studies. That may cause some biases, reducing the power to detect the causal association. However, previous studies have reported that TL measured in blood and lung was correlated, supporting the assumption that our SNPs can predict TL in lung tissue.^39^

In conclusion, our study provides evidence for a possible causal association between telomere length and lung cancer risk in East Asian population, consistent with Western population results. Further studies need to be undertaken to clarify specific mechanisms for telomere in lung cancer carcinogenesis. More efforts also need to combine telomeres with clinical application to improve lung cancer prediction and prevention.

## CONFLICT OF INTEREST

The authors declare no conflict of interest.
