# Supplementary material for: Mendelian randomization study of telomere length and lung cancer risk in East Asian population
Source: Cancer Med. 2019 Oct 11;8(17):7469–76. doi: 10.1002/cam4.2590 (PMC6885879; doi:10.1002/cam4.2590)
Supplement: Supplementary file 1 [file CAM4-8-7469-s001.docx]

**Supplementary Information**

Table S1. Descriptive characteristics of study participants with complete covariate information

Table S2. Previously published variants associated with circulating leukocyte telomere length

Table S3. Associations of telomere length-associated variants and lung cancer risk in NJMU GWAS and FLCCA GWAS

**Table S1.** Descriptive characteristics of study participants with complete covariate information

|  | **NJMU GWAS** | | **FLCCA GWAS** | | **Overall** | |
| --- | --- | --- | --- | --- | --- | --- |
|  | **Cases** | **Controls** | **Cases** | **Controls** | **Cases** | **Controls** |
| All | 2331 | 3077 | 4796 | 3741 | 7127 | 6818 |
| Gender |  |  |  |  |  |  |
| Male | 1711 | 2086 | n/a | n/a | 1711 | 2086 |
| Female | 620 | 991 | 4796 | 3741 | 5416 | 4732 |
| Smoking |  |  |  |  |  |  |
| Nerver | 825 | 1768 | 4796 | 3741 | 5621 | 5509 |
| Smoker | 1506 | 1309 | n/a | n/a | 1506 | 1309 |
| Age |  |  |  |  |  |  |
| < 40 | 71 | 40 | 244 | 278 | 315 | 318 |
| 40-50 | 299 | 349 | 732 | 573 | 1031 | 922 |
| 50-60 | 741 | 1040 | 1432 | 1172 | 2173 | 2212 |
| 60-70 | 780 | 1064 | 1564 | 1206 | 2344 | 2270 |
| >=70 | 440 | 584 | 824 | 512 | 1264 | 1096 |
| Histology |  |  |  |  |  |  |
| Squamous cell carcinoma | 822 | n/a | 660 | n/a | 1482 | n/a |
| Adenocarcinoma | 1304 | n/a | 3469 | n/a | 4773 | n/a |
| Other ^a^ | 205 | n/a | 667 | n/a | 872 | n/a |

**Table S2.** Previously published variants associated with circulating leukocyte telomere length

| SNP | CHR | Position | Gene | Short allele | Long allele^a^ | Reported MAF^b^ | ASN MAF^b^ | Reported estimate^c^ | P | Reference |
| --- | --- | --- | --- | --- | --- | --- | --- | --- | --- | --- |
| rs10936599 | chr3 | 169492101 | TERC | T | C | 0.25 | 0.41 | 0.12 | 2.50×10^-31^ | Codd et al. |
| rs2736100 | chr5 | 1286516 | TERT | A | C | 0.49 | 0.49 | 0.09 | 4.40×10^-19^ | Codd et al. |
| rs7675998 | chr4 | 164007820 | NAF1 | A | G | 0.22 | 0.17 | 0.09 | 4.30×10^-16^ | Codd et al. |
| rs8105767 | chr19 | 22215441 | ZNF208 | A | G | 0.30 | 0.36 | 0.06 | 1.10×10^-9^ | Codd et al. |
| rs755017 | chr20 | 62421622 | RTEL1 | A | G | 0.12 | 0.33 | 0.07 | 6.70×10^-9^ | Codd et al. |
| rs11125529 | chr2 | 54475866 | ACYP2 | C | A | 0.14 | 0.12 | 0.07 | 4.50×10^-8^ | Codd et al. |
| rs4387287 | chr10 | 105677897 | OBFC1 | A | C | 0.08 | 0.18 | 0.12 | 3.90×10^-9^ | Levy et al. |
| rs3027234 | chr17 | 8136092 | CTC1 | T | C | 0.23 | 0.06 | 0.06 | 2.30×10^-8^ | Mangino et al. |
| rs412658 | chr19 | 22151280 | ZNF676 | C | T | 0.36 | 0.39 | 0.05 | 9.80×10^-9^ | Mangino et al. |

^a^ Long allele is allele associated with longer telomere length;

^b^ MAF, minor allele frequency;

^c^ Report estimate is reported in telomere kb per long allele.

**Table S3.** Associations of telomere length-associated variants and lung cancer risk in NJMU GWAS and FLCCA GWAS

| SNP | NJMU GWAS | | | FLCCA GWAS | | | Het *P* ^h^ |
| --- | --- | --- | --- | --- | --- | --- | --- |
|  | OR | 95%CI | *P* | OR | 95%CI | *P* |  |
| rs10936599 | 1.08 | (1.00,1.18) | 0.062 | 1.08 | (1.01,1.15) | 0.015 | 0.929 |
| rs2736100 | 1.21 | (1.11,1.32) | 9.53×10^-6^ | 1.38 | (1.30,1.47) | 9.96×10^-25^ | 0.012 |
| rs7675998 | 1.10 | (0.99,1.23) | 0.074 | 1.07 | (0.98,1.16) | 0.118 | 0.611 |
| rs4387287 | 1.01 | (0.90,1.13) | 0.861 | 0.98 | (0.91,1.07) | 0.703 | 0.714 |
| rs8105767 | 1.02 | (0.93,1.12) | 0.652 | 1.02 | (0.96,1.09) | 0.462 | 0.952 |
| rs755017 | 0.93 | (0.85,1.01) | 0.074 | 1.00 | (0.94,1.06) | 0.887 | 0.172 |
| rs11125529 | 0.94 | (0.85,1.05) | 0.270 | 1.10 | (1.01,1.19) | 0.022 | 0.024 |
| rs3027234 | 0.95 | (0.76,1.18) | 0.627 | 1.16 | (1.00,1.36) | 0.054 | 0.130 |
| rs412658 | 0.98 | (0.90,1.07) | 0.679 | 1.05 | (0.99,1.12) | 0.123 | 0.216 |
| Aggregate test^a^ |  |  | 1.21×10^-4^ |  |  | 2.75×10^-23^ |  |
| Genetic risk score^b^ | 1.58 | (1.10,2.28) | 0.013 | 2.59 | (1.98,3.39) | 3.76×10^-12^ |  |
| MR(IVW)^c^ | 1.65 | (1.13,2.41) | 9.51×10^-3^ | 2.74 | (2.07,3.61) | 9.99×10^-13^ |  |
| Heterogeneity^d^ |  |  | 1.80×10^-3^ |  |  | 5.17×10^-13^ |  |
| Genetic risk score exclude rs2736100^e^ | 1.15 | (0.77,1.72) | 0.488 | 1.59 | (1.19,2.14) | 1.19×10^-3^ | 0.201 |
| LUAD Genetic risk score^f^ | 2.18 | (1.42,3.35) | 3.40×10^-4^ | 2.83 | (2.11,3.80) | 4.27×10^-12^ | 0.324 |
| LUSC Genetic risk score^g^ | 1.01 | (0.60,1.71) | 0.970 | 1.92 | (1.13,3.25) | 0.015 | 0.092 |

^a^ Aggregate test is a log likelihood ratio test comparing a model having all telomere length-associated SNPs and covariates with a null model;

^b^ Genetic risk score ORs refer to a 1-kb increase in telomere length;

^c^ Inverse-variance weighted Mendelian randomization estimate for a 1-kb increase in telomere length;

^d^ Test for significant heterogeneity across the nine SNP instruments used in the Mendelian randomization analysis;

^e^ Genetic risk score ORs refer to a 1-kb increase in telomere length after excluding rs2736100;

^f^ Genetic risk score ORs refer to a 1-kb increase in telomere length for lung adenocarcinoma;

^g^ Genetic risk score ORs refer to a 1-kb increase in telomere length for lung squamous cell carcinoma;

^h^ *P* value for heterogeneity test between two studies.
